# Applicability of a single camera-based catheter navigation system using teeth arch as an anatomical landmark for superselective intraarterial infusion in advanced oral cancer treatment

**DOI:** 10.1007/s11517-021-02326-w

**Published:** 2021-02-16

**Authors:** Ken Yanagida, Takashi Ohya, Junchen Wang, Toshinori Iwai, Toshiharu Izumi, Etsuko Kobayashi, Ichiro Sakuma, Kenji Mitsudo

**Affiliations:** 1 Department of Oral and Maxillofacial Surgery, Yokohama City University Graduate School of Medicine, 3-9 Fukuura, Kanazawa-ku, Yokohama, 236-0004 Japan; 2 School of Engineering, The University of Tokyo, Tokyo, Japan; 3 School of Mechanical Engineering and Automation, Beihang University, Beijing, China; 4 Department of Radiology, Yokohama City University Hospital, Yokohama, Japan

**Keywords:** Catheter, Oral cancer, Three dimensional, Fiducial markers

## Abstract

Superselective intraarterial infusion chemoradiotherapy is a modality of oral cancer therapy in which the artery feeding the tumor is catheterized. 3D information about the carotid artery is required to enable the surgeon to judge whether to advance, retract, or rotate the catheter. For this purpose, we proposed and conducted a model experiment to assess a new method of catheterization that applies a tracking system using registration with a monocular camera using the maxillary arch as the anatomical landmark. In this method, the preoperative 3D computer tomography angiographic image of the carotid artery that the catheter will be passed through is overlaid on the 2D video image. The mean TRE was 0.96 ± 0.36 mm and 0.88 ± 0.31 mm and 1.12 ± 0.46 mm when images were registered with the anterior and posterior teeth as the landmarks, respectively; the difference was not significant (*p* = 0.21). This tracking system that enables markerless registration simply by taking images of the maxillary anterior teeth with a single camera was convenient and effective for catheterization. In this study, we propose the new application of this tracking system and a novel method of catheterization for superselective intraarterial infusion chemoradiotherapy for oral cancer.

**Figure:**
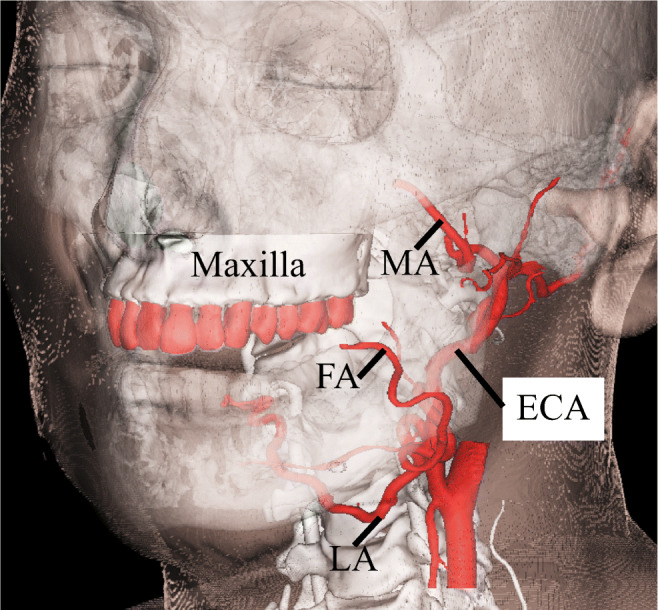
In retrograde superselective intraarterial catheterization, a catheter is inserted into a tumor-feeding artery originating from the external carotid artery (ECA) (the lingual artery [LA], facial artery [FA], or maxillary artery [MA]). Because the maxillary dentition is located near the external carotid artery, we focused on real-time markerless registration using maxillary dentition fixed to the skull.

In retrograde superselective intraarterial catheterization, a catheter is inserted into a tumor-feeding artery originating from the external carotid artery (ECA) (the lingual artery [LA], facial artery [FA], or maxillary artery [MA]). Because the maxillary dentition is located near the external carotid artery, we focused on real-time markerless registration using maxillary dentition fixed to the skull.

## Introduction

Retrograde superselective intraarterial infusion has superior antitumor effects for advanced oral cancer [1–3]. This therapeutic method involves inserting a catheter into an artery located anteriorly or posteriorly to the ear and placing the catheter tip to the artery feeding the oral cancer (hereinafter, catheterization) [2–8]. This method of catheterization uses two-dimensional (2D) C-arm X-ray imaging to locate the catheter tip. Operative time for this surgery can become long in cases in which guiding the catheter tip to the target vessel is challenging. Such prolonged surgeries increase X-ray exposure for both the surgeon and the patient and the quantity of the contrast agent used, thereby increasing the burden for both the surgeon and patient. To resolve these issues, the recent methods of navigation systems for catheterization propose using 3D data [7]. While the ultimate location of the catheter tip is verified by X-ray imaging as has been conventionally performed, the recent navigation systems effectively use preoperative computer tomography angiography (CTA) imaging to guide the catheter close to the target vessel. However, while there are several existing methods of registration in conventional navigation systems, none is without weaknesses that have prevented them from being used more extensively in clinical practice. For example, the reference stent with a marker demands work and time for preparation. The attachment of reference markers to the face involves problems associated with facial skin shift such as incidental errors which, at times, can be significant. Moreover, skin shift is a problem with noncontact registration methods that use infrared laser to obtain groups of position coordinates on the facial surface [9]. This study thus tested the adaptability of catheterization in which the imaging of the maxillary teeth with a single camera allows tracking with the maxillary teeth as a landmark to overlay the preoperative CTA image with the patient’s video image data [10].

The registration system applied in this study uses the morphology of the dental arch as a landmark in obtaining 3D data from a single camera that projects 3D CTA images on a 2D display. Though the two images could be superimposed with sufficient accuracy to confirm adequate tracking at an intuitive level using the existing methods, it was difficult to display this quantitatively and was therefore an area that required improvement.

This study aims to propose a method to assess registration with this single camera and the accuracy of the overlaying system (hereinafter, tracking system) to superimpose it with the video image and to test the feasibility of newly expanding its application to encompass catheterization. An optical position locating device with small error was used for this tracking system; therefore, the model and virtual target points must be pointed and measured with the tip of the location measurement probe by a human to assess accuracy. Although this is possible with a model, it is difficult to do on a virtual target point. To resolve this issue, the error was measured by setting a target point on the plane of the model perpendicular to the camera’s optical axis and virtual plane positioned parallel to the model plane.

One of the limitations of this study is that it measures error after excluding depth information; that is, 3D measurements of error are not taken. However, this limitation will be compensated by testing that the spread is minimal and will not cause anatomical issues. Data will be measured at the various bifurcations and measured in 10 patients to demonstrate that the lack of information about depth does not impact the results.

The second limitation of this study is that it did not assess the catheter navigation system. For actual application, it would be necessary to load a magnetic sensor on the catheter to assess accuracy. However, this study focused on the application of a tracking system with a single camera. Therefore, an optical position locating device that allows stable 3D error measurement was used for the accuracy test in this study.

There are multiple benefits to having a CTA image that is tracked and displayed just by taking the image of the teeth with a single camera. First, because teeth are hard tissues, it is not affected by skin shifting that can occur because of swelling or deformation, which makes reregistration unnecessary. Second, easy setup allows navigation surgery to be immediately started, which is a tremendous advantage for medical staff. This study seeks to propose this as a method to enable simpler surgical navigation than conventional methods and demonstrate its applicability for the overlay presentation of the carotid artery for catheterization.

## Methods

This study was approved by the Yokohama City University Ethics Committee (No. B150801012) and conducted in conformance to the guidelines of the Declaration of Helsinki.

### Patient selection

Ten patients with oral cancer with 14 maxillary teeth and without metal restorative materials were selected. Nine were men, and one was a woman, and their median age was 66 (range 39–84) years. Informed consent was obtained from the patients. The CTA was taken in these patients before administering superselective chemotherapy to map their external carotid artery (ECA) and branches.

### Computer tomography angiography

A 64-slice CT scanner (Aquilion 64, Toshiba Medical Systems, Tokyo, Japan) scanner was used. An automatic power injector was used to inject a nonionic contrast agent (100 mL) at a rate of 4.0 mL/s from the antecubital vein. The bolus tracking technique was used to individually select the scan delay in the arterial phase. Low-dose scans were repeatedly taken with a delay of 8.00 s at the level central to the carotid bifurcation. To measure the bolus arrival time, the region of interest was selected in the common carotid artery. The scanning procedure was started automatically once arrived at the 90 Hounsfield unit enhancement level. The scanning volume for the arterial phase included the lower rim of the thyroid cartilage/lower rim at the C6 level, as well as the supraorbital margin.

The images were taken in the following settings: 120 kV, 250 mA, 64 × 0.5 mm slice collimation, table velocity 20.5 mm/rotation (pitch = 0.641), rotation time = 0.75 s, resolution matrix = 512 × 512 pixels, and 1.0 mm slice thickness. CTA data was exported in the digital imaging and communication in medicine (DICOM) format.

### Phantom fabrication

**Fig. 1:**
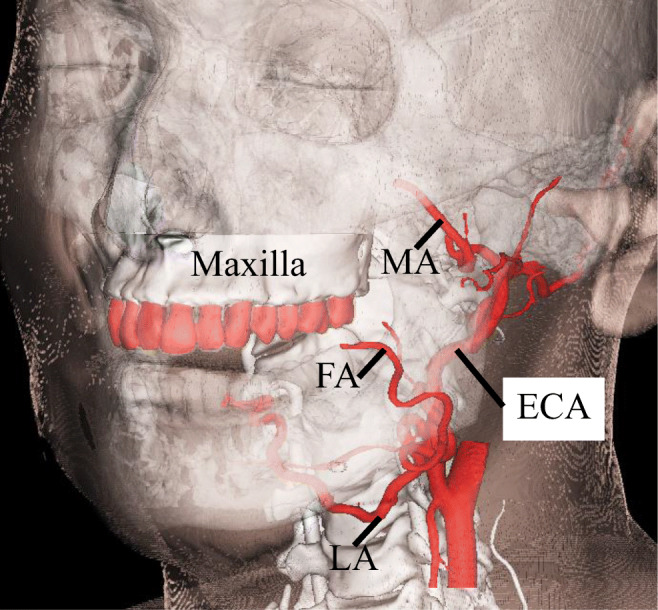
Clinical anatomy. Maxilla teeth are close to tumor-feeding arteries, including the LA, FA, and MA. These arteries arise from the ECA. *LA* lingual artery, *FA* facial artery, *MA* maxillary artery, *ECA* external carotid artery

**Fig. 2:**
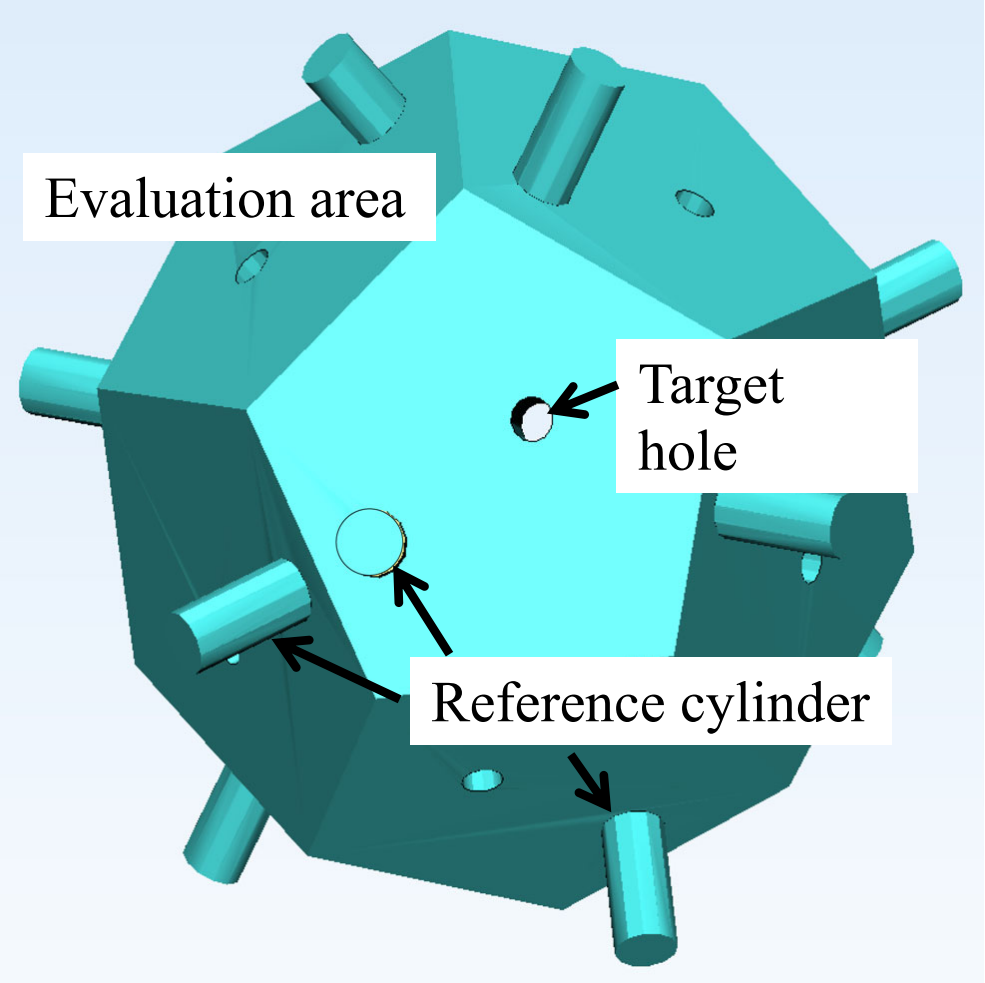
Regular dodecahedron in the software. A target hole (1 mm in diameter) is set on the pentagon plane, and a reference cylinder (3.0 mm in length and 1.5 mm in diameter) is set perpendicular to the pentagon plane. This pentagon plane has sides 7 mm in length

**Fig. 3:**
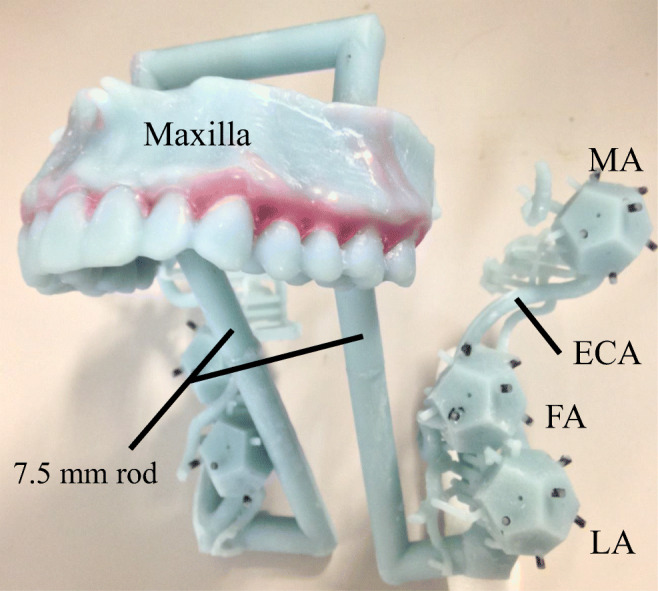
Maxilla-ECA phantom. There are three bifurcations between the ECA and tumor-feeding arteries, including LA, FA, and MA. The reference cylinder is shaded black. *LA* lingual artery, *FA* facial artery, *MA* maxillary artery, *ECA* external carotid artery

CTA data from 10 patients was entered into the software (Mimics ver.16.0; Materialize, Leuven, Belgium) in DICOM format. Data on the maxilla and bilateral external carotid arteries (ECA) were segmented and connected with rods (7.5 mm diameter). To assess registration error, the center of the regular dodecahedron model was placed at the bifurcations with the tumor-feeding arteries, including the bifurcations of the ECA and lingual artery (LA), facial artery (FA), and maxillary artery (MA) (Fig. 1). The faces of the regular dodecahedron were equilateral pentagons with 7.0 mm sides. A target hole (1.0 mm diameter) was set on the equilateral pentagonal faces and used as a point for measurement by the optical tracker (Polaris SPECTRA, Northern Digital Inc., Ontario, Canada). A reference cylinder (3.0 mm long, 1.5 mm diameter) was further placed perpendicularly to the pentagonal faces (Fig. 2). Because the target is pointed by the measurement probe while actually viewing the 2D image, the camera was placed such that any registration errors, with the exception of those that involve depth information, could be recognized. That is, the experiment was conducted with the camera’s optical axis adjusted as if the reference cylinder would be viewed from above. The angle was verified by confirming that the sides of neither the reference cylinder of the model or the overlaid image were invisible. Assessments were thoroughly made by repeating the same on various individuals and at various arterial bifurcations. Data were exported in standard triangulated language (STL) format. After importing STL data, a 3D printer (Digital Wax 028J Plus, DWS s.r.l., Vicenza, Veneto, Italy) was used to create 10 maxillary ECA phantoms (Fig. 3). The phantoms were fabricated by a special laser that hardens a photo-curable resin. The direct molding resin-Thermal DM210 was used, which was a nanofilled ceramic, including liquid silicone and vulcanized rubber. The scanning method was galvanometer and a 3D printer resolution of 40 μm.

### Obtaining phantom image data

To eliminate error originating in deformations that occur in the 3D manufacturing process, the maxillary ECA phantom was scanned using the same CT system. DICOM data was segmented and converted into STL format using Mimics.

### Markerless registration

Markerless registration was performed using a novel 3D–2D matching method that involves building a similarity-based hierarchical aspect graph offline and matching it with a live video for pose estimation online [10, 11]. The hierarchical aspect graph was built online using the cascade method across differing image levels and iteratively combining similar virtual views offline. The virtual and real cameras were calibrated and set in various positions to take images of STL and actual model. The degree of similarity between the virtual and actual images was assessed by averaging the inner product of the contour vector and the corresponding image gradient [12]. The following metric is used to measure the similarity between the shape *E*_2D_ and the search image *I* (*x*_*i*_, *y*_*i*_):

1 $$ s\left({E}_{2D},I\right)=\frac{1}{N}\sum \limits_{i=1}^N\frac{\ \left\langle \nabla I\left({x}_i{y}_i\right),{d}_i\right\rangle \kern0.75em }{\left\Vert \nabla I\left({x}_i,{y}_i\left.\Big)\ \right\Vert \right.\left\Vert {d}_i\right\Vert \right.} $$

where <,> denotes dot product, and ∇*I* (*x*_*i*_, *y*_*i*_) is the image gradient at (*x*_*i*_, *y*_*i*_). Sample data for various views were obtained in advance because the 3D posture could not be directly optimized by Eq. (1). In the online step, the hierarchical aspect graph was crossed to identify the pose with the highest degree of similarity with the virtual image. The iterative closest point algorithm was selected to further narrow down the searched pose [13], and a graphics processor was selected for high-speed calculation.

### Phantom experiment

**Fig. 4:**
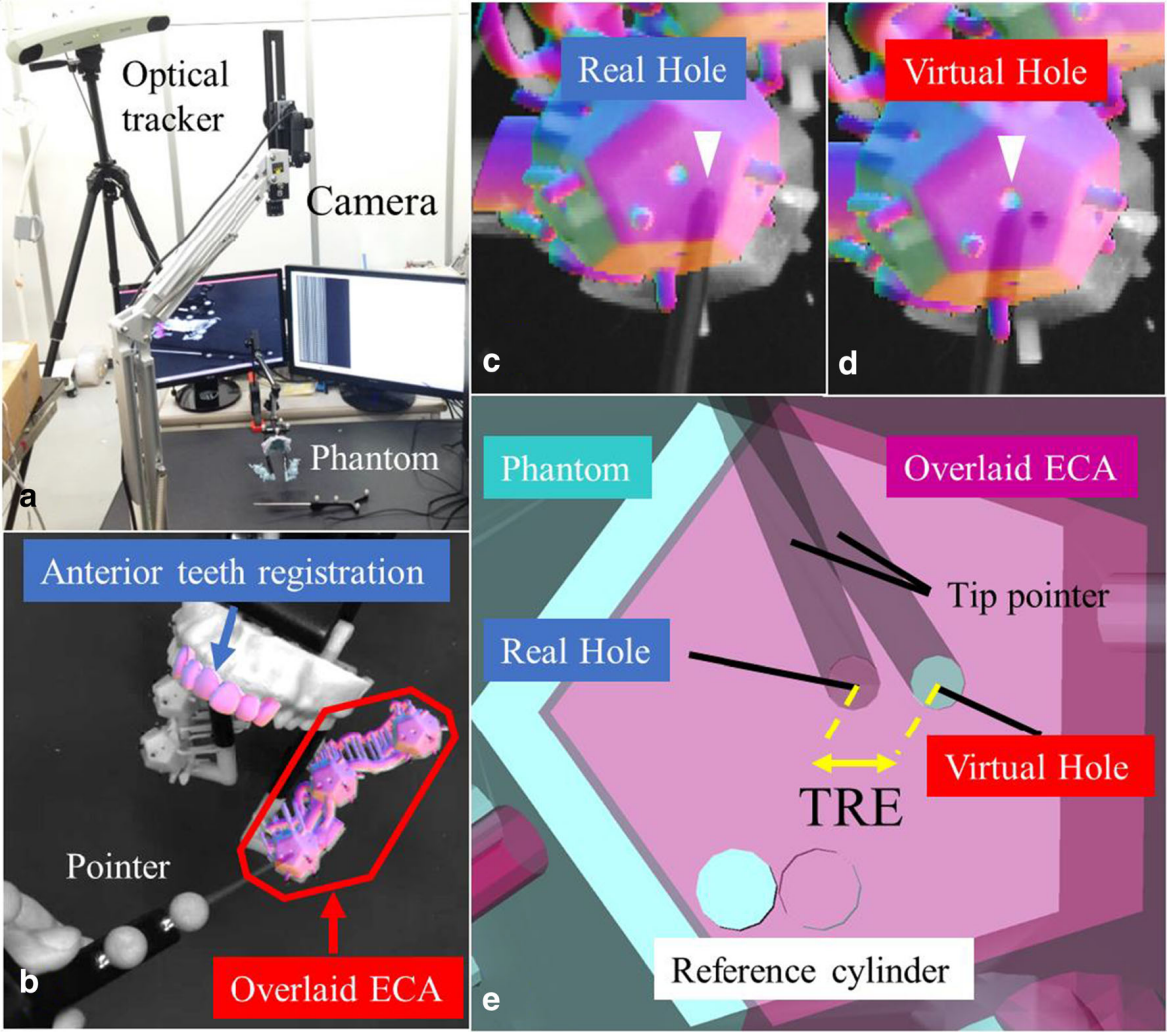
**a** Experimental setup. The distance from the camera to the phantom is ~60 cm. The phantom and camera are fixed to table with a jig, respectively. **b** Maxillary anterior teeth registration and overlay of the left external carotid artery (ECA). **c** The tip of the pointer (arrow) is inserted into the phantom real hole (arrowhead). **d** The tip of the pointer (arrow) is placed at the overlaid virtual hole (arrowhead). **e** Magnified image of the target registration error (TRE) between the phantom real hole and overlaid virtual hole

The various maxillary ECA phantoms were placed beneath the single monochrome camera (UI-3370CP-M-GL, IDS Imaging Development Systems GmbH, Obersulm, Germany) (Fig. 4a). The camera’s imaging resolution was set at 2048 × 2048 pixels. Camera calibration was performed to eliminate distortion [14]. An optical tracker was selected to assess the target registration error (TRE). The upper arch was registered for 20–50 s on the offline camera image, and then the ECA overlay was performed within 1 s on the online video (Fig. 4b). On the online movie, the tracking-learning-detection framework was used within 512 × 512 pixels of the feature point [15]. This framework was intended to reduce calculation time and to eliminate false registration. All core algorithms were written in C++, and OpenGL Shading language (GLSL 4.50) was used for graphic rendering and parallel computing.

The personal computer used in this study had an Intel Core i7-4820K CPU (3.7 GHz; Intel Corp., Santa Clara, California) and an NVIDIA GeForce GTX TITAN GPU (NVIDIA Corp., Santa Clara, CA) and was running Microsoft Windows 7 Professional operating system (Microsoft Corp., Redmond, Washington).

### Target registration error assessment

Because the system we are proposing uses only 2D images (that is, data without depth information), an equilateral pentagon phantom was used to assess absolute error. The absolute distance between the target hole (real hole) (Fig. 4c) and the virtual hole (Fig. 4d) was measured with an optical tracker.

**Fig. 5:**
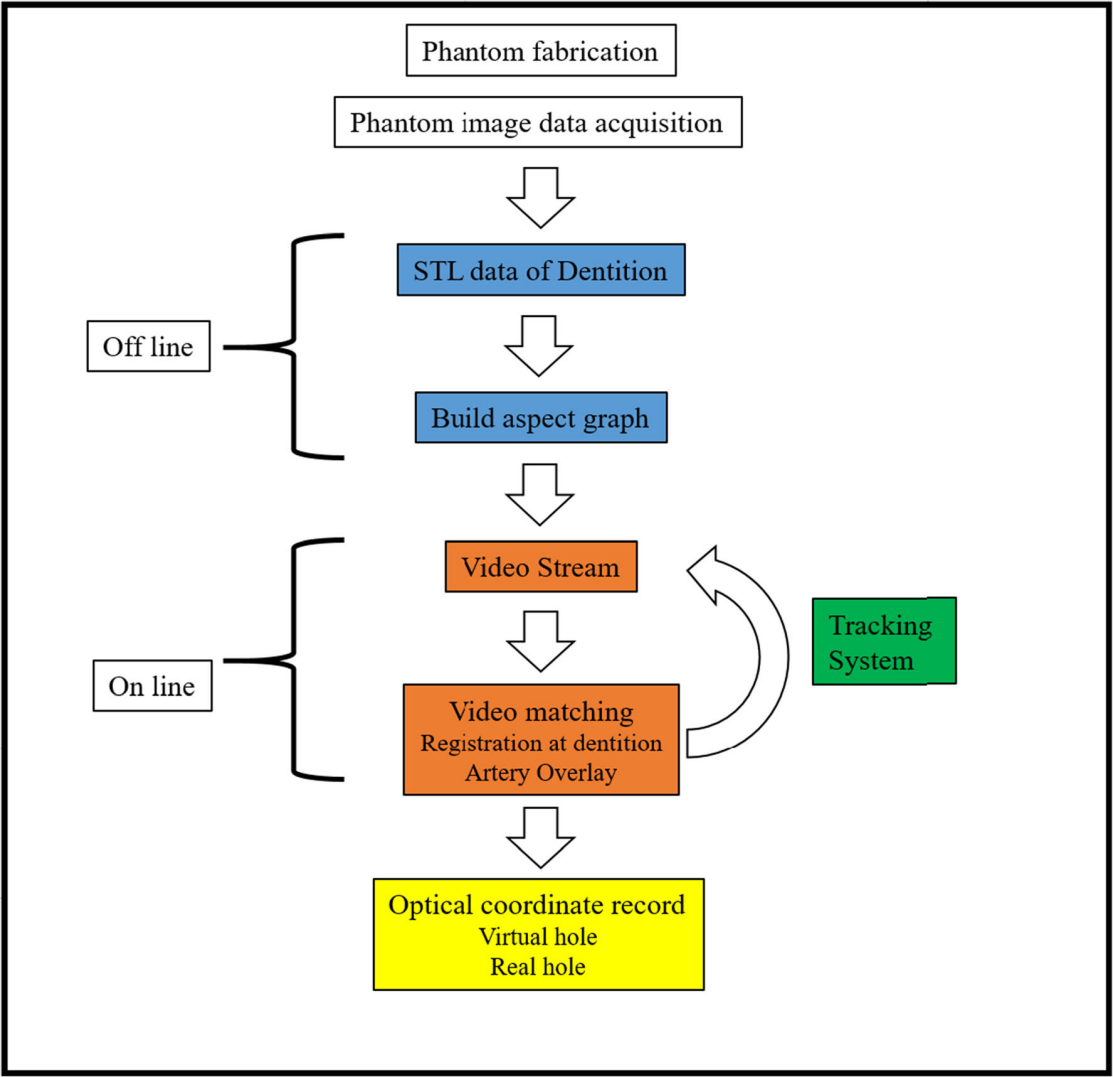
Operating sketch of the phantom experiment

The registration of the maxillary arch was performed with two patterns: with the anterior (4 incisors and 2 canines) and posterior teeth (2 premolars and 2 molars). After performing registration of the maxillary teeth and ECA overlay, the tip of the measurement probe of the optical position locating device was placed at the center of the real hole on the pentagon (Fig. 4c) and then on the center of the virtual hole (Fig. 4d). Next, the distance between the virtual and real holes was calculated (Fig. 4e). The TRE was measured three times at each of the bifurcations of the six branches (bilateral LA, FA, and MA) in 10 patients. Figure 5 shows the steps from fabricating the phantom to the assessment of the TRE.

### Statistical analysis

The Student *t* test was used for the statistical comparison of the TRE of the maxillary anterior and posterior teeth registrations. IBM SPSS Statistics 21 for Windows (IBM Corp., Armonk, New York) was used for all statistical analysis where *p* < 0.05 was considered statistically significant.

## Results

**Fig. 6:**
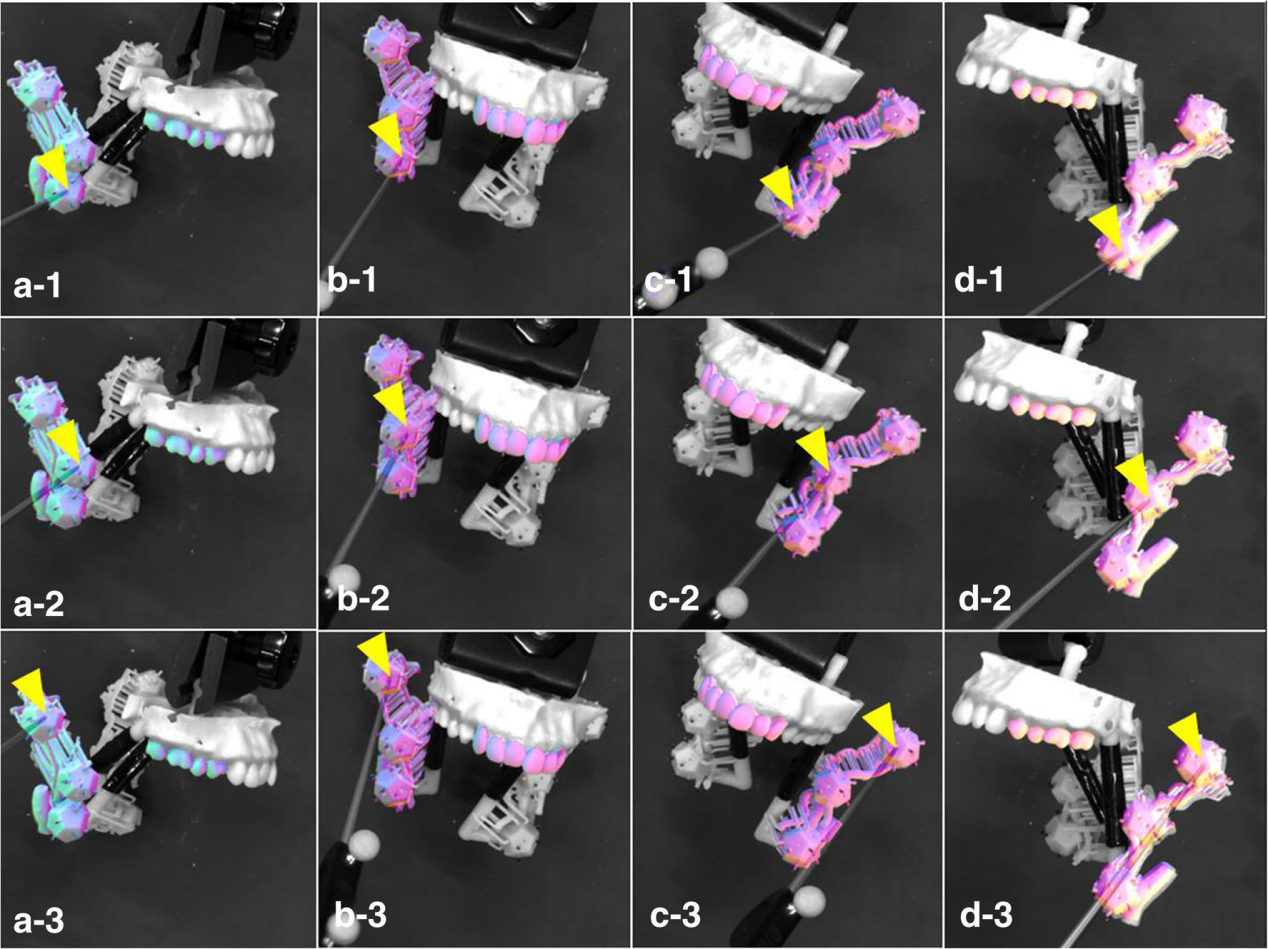
Registration teeth and overlaid ECA patterns. **a**–**c** indicate the registration teeth and overlaid ECA patterns. Numbers 1–3 indicate the measurement bifurcation points. In each image, the tip of the pointer is indicated with a yellow triangle. **a** Right posterior teeth registration and right ECA overlay. **b** Anterior teeth registration and right ECA overlay. **c** Anterior teeth registration and left ECA overlay. **d** Left posterior teeth registration and left ECA overlay. (1) LA bifurcation measurement. (2) FA bifurcation measurement. (3) MA bifurcation measurement. *LA* lingual artery, *FA* facial artery, *MA* maxillary artery, *ECA* external carotid artery

**Fig. 7:**
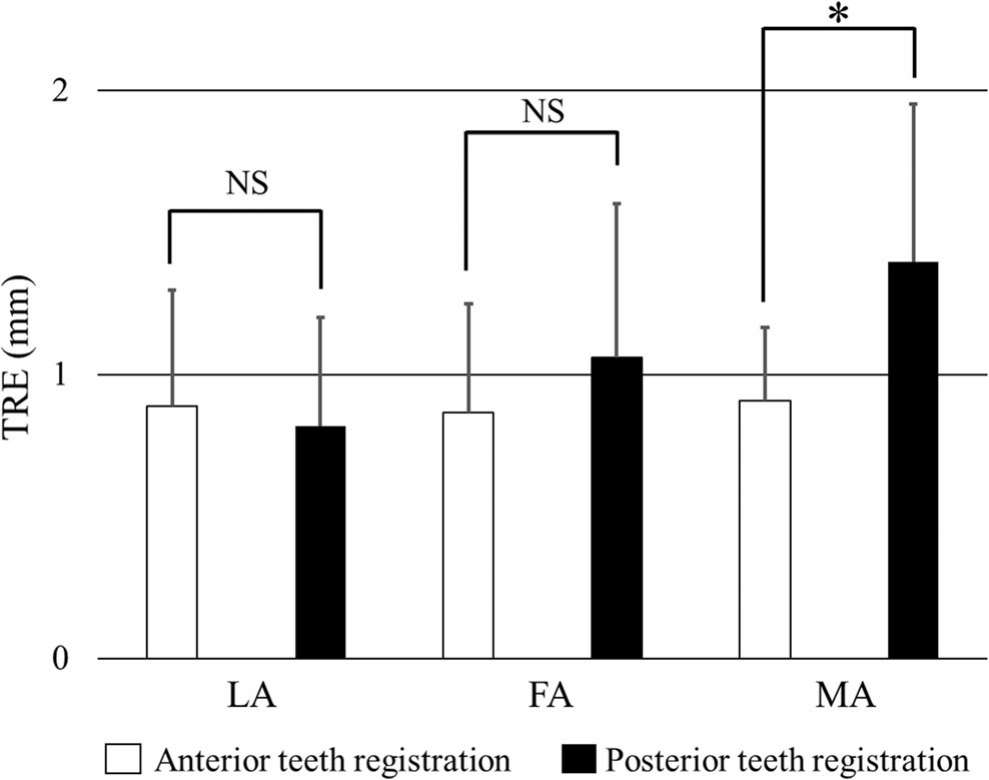
Target registration error (TRE) after anterior and posterior teeth registrations at each bifurcation point. *LA* lingual artery, *FA* facial artery, *MA* maxillary artery, *NS* not significant, *p < 0.05

The mean TRE (RMS ± standard deviation) of the posterior maxillary teeth was 1.12 ± 0.46 mm (Fig. 6a-1, 2, 3, d-1, 2, 3), and the mean TRE of the anterior maxillary teeth was 0.88 ± 0.31 mm (Fig. 6b-1, 2, 3, c-1, 2, 3). There was no significant difference in the TRE between the two references: the anterior and posterior teeth alignment. The mean TRE for the measured LA, FA, and MA branches after registering the maxillary posterior teeth were 0.82 ± 0.38 (Fig. 6a-1, d-1), 1.06 ± 0.54 (Fig. 6a-2, d-2), and 1.40 ± 0.55 mm (Fig. 6a-3, d-3), respectively. The mean TRE for the measured LA, FA, and MA branches after registering the maxillary anterior teeth were 0.89 ± 0.41 (Fig. 6b-1, c-1), 0.87 ± 0.38 (Fig. 6b-2, c-2), and 0.91 ± 0.25 (Fig. 6b-3, c-3), respectively. A significant difference was reported in the TRE between the anterior and posterior teeth for the bifurcation with the MA only (Fig. 7). The results of this study are presented as both RMS and mean values (Table 1).

**Table 1:** Target registration error. The left row indicates the evaluation area. The registration sites and the results for RMS ± std (mm) and mean ± std (mm) are shown

	Anterior teeth registration		Posterior teeth registration	
	RMS ± std (mm)	Mean ± std (mm)	RMS ± std (mm)	Mean ± std (mm)
LA	0.89 ± 0.41	0.74 ± 0.41	0.82 ± 0.38	0.71 ± 0.37
FA	0.87 ± 0.38	0.76 ± 0.39	1.06 ± 0.54	0.86 ± 0.52
MA	0.91 ± 0.25	0.84 ± 0.26	1.40 ± 0.55	1.19 ± 0.52
Total	0.88 ± 0.31	0.78 ± 0.33	1.18 ± 0.46	0.92 ± 0.44

## Discussion

This study tested whether a tracking system using a single camera can be applied for catheterization in the carotid artery region. The tracking system was special in that it superimposed 3D images of the maxillary arch obtained from a single camera with 3D CTA images taken preoperatively and projected them on a 2D display. Furthermore, there was a distance of 10 cm between the maxillary teeth, which were used as the landmark for registration and the target, i.e., the carotid arteries. Given these special conditions, a novel method was required to be proposed to make measurements for an accuracy assessment, which was the purpose of this study. We fabricated a phantom for assessment and tested the proposed accuracy assessment method that excluded depth information. This is a limitation of this system itself that displays an overlay image on 2D images, which inevitably lacks the capacity to accurately capture depth, which necessitates a discussion on how this tracking system can be used in clinical practice. First, the single camera setup in clinical use will not be much different from the setup in this experiment. In other words, the relationship between the position of the camera for capturing the anterior teeth and the position of the carotid arteries is more or less the same between individuals. Furthermore, this method can be considered effective as long as it provides information for the surgeon to determine whether to advance, retract, or rotate the catheter. In that sense, the error on the plane perpendicular to the optical axis of the single camera is ~1 mm, even if the depth information of the optical axis is missing. Because the diameter of the carotid artery is ~3–5 mm, the level of effectiveness is adequate. This system may not be adaptable for all types of surgeries for the head and neck region. For example, it will not be effective for determining the accuracy of the angle of dental implants into the jawbone. It is important to reiterate that the results of this study only indicate that the system is suitable for catheterization in the head and neck region. The differences in error at the various bifurcations of the artery were only ~0.6 mm, which demonstrates that the error is insignificant even when the image of the referential maxillary arch is taken from slightly different angles with a single camera. Furthermore, there was little spread in errors between individuals (~0.3 mm). This suggests that, in the carotid artery, which is the vessel treated by this technique, there is little anatomical variation in the bifurcations, confirming that adequate reproducibility has been achieved.

However, in this study, optical tracker is used for accuracy assessment of the tracking system using a single camera. While an optical tracker has many strengths such as minimizing error and generating stable measurements, it has a weakness: in the face of an object that obstructs the light, measurement becomes impossible. Because catheter navigation systems actually measure the sensor that is inserted into the body, they are only completed by combining a magnetic position sensor.

Seeberger et al. [16] used a skull phantom and maxillary model to assess the TRE of an electromagnetic tracking navigation system under various conditions (Aurora, Northern Digital Inc., Ontario, Canada). The mean TRE of five-point (five titanium screws on the maxillary arch registration template) and six-point registration (five titanium screws and an additional marker at the temporal cranial base) under experimental and operating room conditions were 1.85 ± 0.69 and 0.69 ± 0.35 mm and 2.10 ± 0.86 and 1.03 ± 0.53 mm, respectively. Our previous study [17] reported that the mean TRE of a magnetic tracking catheter navigation system following point-based registration was 1.80 ± 0.85 mm under experimental room conditions. These results suggest that while combining a magnetic tracking system to the tracking system proposed in this study might increase error compared to the current results, it would still be well within the range of errors that would be considered an effective system.

Markerless registration in maxillofacial surgical navigation should be based on hard tissues that do not deform. Selecting soft tissues as the landmark involves the risk of skin shift, which can incidentally cause significant errors. This was why we focused on the maxillary teeth as the target for automatic markerless registration tested in this study. Unlike general oral or maxillofacial surgery, the ECA, which is the target of catheterization, is far from the maxillary teeth. However, the distance between the ECA and the maxillary teeth is only ~10 cm; therefore, we predicted that it would not involve major errors. Indeed, our study resulted in a mean TRE of anterior and posterior maxillary teeth registration of 0.88 ± 0.31 and 1.12 ± 0.46 mm, respectively, demonstrating that the application of this catheter navigation system is feasible. Moreover, there was no significant difference in errors whether the anterior or posterior teeth were used as the landmark for registration; therefore, we were able to understand that the anterior teeth would be a more practical landmark in clinical use because it is easier to take images of the anterior teeth with a camera. In this study, we assessed the TRE in automatic markerless registration under ideal conditions, i.e., in patients with 14 maxillary teeth with few or no metallic artifacts that would be easily detected on CT. For major metal artifacts on CT, the integration of optically scanned dental models and 3D-CT models on software could be a good alternative procedure [18, 19]. In the future, we plan to develop these results into a clinical case study.

Intraoperative vascular deformation is another challenge in catheter navigation systems. In our previous study, we reported that while the shift of peripheral arteries such as the superficial temporal artery is minor, the shift tends to be major in the central ECA [20]. Though it may be possible to prevent vascular deformation using a thermoplastic facial mask used for registration in radio frequency thermocoagulation treatment [21], this would require taking additional CTA with the thermoplastic facial mask. An alternative to this is the detection of intraoperative ECA deformations by applying 3D ultrasound-guided catheter testing, which we have proposed in a previous study [22]. In the future, we plan to study the combination of the present tracking system and ultrasound guidance to distinguish shifts of the ECA and common carotid artery.

Our ultimate goal is to combine a tracking system that tracks a small magnetic center embedded in the catheter with the present tracking system to make easy, real-time registration possible to perfect a novel navigation system that allows quick, convenient, and stress-free testing for medical workers.

## Conclusions

Our findings suggested the effectiveness of a tracking system that tracks and displays CTA images of the carotid artery on video image simply by capturing the anterior or posterior maxillary teeth images with a single camera applied for catheterization. This system is clinically effective in that capturing the maxillary anterior teeth images with a single camera allows for markerless registration for catheterization within a relatively small area of the head and neck region.

## Abbreviations

CTA: Computed tomography angiography
ECA: External carotid artery
TRE: Target registration error
STA: Superficial temporal artery
LA: Lingual artery
FA: Facial artery
MA: Maxillary artery
DICOM: Digital imaging and communication in medicine
STL: Standard triangulated language
